# Skeleton extraction and pruning point identification of jujube tree for dormant pruning using space colonization algorithm

**DOI:** 10.3389/fpls.2022.1103794

**Published:** 2023-01-19

**Authors:** Yuxing Fu, Yuyang Xia, Huiming Zhang, Meng Fu, Yong Wang, Wei Fu, Congju Shen

**Affiliations:** ^1^ School of Mechanical and Electrical Engineering, Hainan University, Haikou, China; ^2^ School of Information and Communication Engineering, Hainan University, Haikou, China; ^3^ Mechanical Equipment Research Institute, Xinjiang Academy of Agricultural and Reclamation Sciences, Shihezi, China

**Keywords:** dormant pruning, space colonization algorithm, skeleton, pruning point identification, jujube tree

## Abstract

The dormant pruning of jujube is a labor-intensive and time-consuming activity in the production and management of jujube orchards, which mainly depends on manual operation. Automatic pruning using robots could be a better way to solve the shortage of skilled labor and improve efficiency. In order to realize automatic pruning of jujube trees, a method of pruning point identification based on skeleton information is presented. This study used an RGB-D camera to collect multi-view information on jujube trees and built a complete point cloud information model of jujube trees. The space colonization algorithm acts on the global point cloud to generate the skeleton of jujube trees. The iterative relationship between skeleton points was represented by constructing a directed graph. The proposed skeleton analysis algorithm marked the skeleton as the trunk, the primary branches, and the lateral branches and identified the pruning points under the guidance of pruning rules. Finally, the visual model of the pruned jujube tree was established through the skeleton information. The results showed that the registration errors of individual jujube trees were less than 0.91 cm, and the average registration error was 0.66 cm, which provided a favorable database for skeleton extraction. The skeleton structure extracted by the space colonization algorithm had a high degree of coincidence with jujube trees, and the identified pruning points were all located on the primary branches of jujube trees. The study provides a method to identify the pruning points of jujube trees and successfully verifies the validity of the pruning points, which can provide a reference for the location of the pruning points and visual research basis for automatic pruning.

## 1 Introduction

Jujube is a cash crop with Chinese characteristics, which is rich in vitamin C and other nutrients needed by humans. It has high nutritional value and medical value. Chinese jujube planting area has accounted for 99% of the world’s total (Fu et al., 2020). Dormant pruning is a seasonal and labor-intensive orchard management work. With the shortage of labor and the rise of labor costs, the cost of pruning exceeds 20% of the management cost of the whole jujube garden (Crassweller et al., 2020). Pruning can remove low-yield branches, reduce canopy density, improve lighting conditions, and improve the quality of jujube (Kolmanič et al., 2021). Mechanical equipment for mass pruning has been widely studied over the last few decades (Mika et al., 2016). Among them, the geometric pruning equipment has high efficiency and is suitable for rough pruning of fruit trees, but non-selective pruning operations easily cause false and missed pruning of branches (Ma et al., 2021a). Therefore, automatic pruning could effectively avoid the quality degradation caused by incorrect pruning by selecting appropriate pruning points (He and Schupp, 2018). Robotic selective pruning has achieved remarkable results on grapes and apples, but the relevant technologies are still in the experimental stage (Botterill et al., 2017; Zahid et al., 2020; Zahid et al., 2021). The automatic pruning technology of open-center jujube trees with variable structures needs to be studied in-depth.

Identifying potential pruning points was critical to the success of automatic pruning, which could guide pruning actuators to selectively prune unprofitable and diseased branches. Over the last decade, two-dimensional (2D) images and three-dimensional (3D) point clouds have been widely used to detect tree structures (Digumarti et al., 2018). Zhang et al., 2018; Majeed et al. (2020) used the Seg-Net depth learning network to identify trunks and branches of trunk-shaped apple trees and analyzed the segmentation accuracy of apple trees. Karkee et al. (2015) obtained point cloud information on the leafless trunk-shaped apple tree through the built visual system and used specific neighborhood rules to segment the branches. The recognition accuracy of pruned branches was 77%. The reconstruction process of this study was complex, and the recognition accuracy needs to be further improved. Ma et al. (2021b) used the Spg-Net depth learning network to segment jujube branches and established a linear regression model between the ground truth and the predicted value of the number of pruned branches. The above research focused on the segmentation of branches and trunks in the image and did not explore the location of pruning points. Wang et al. (2022) used the Mask R-CNN instance segmentation network to segment branches and bifurcate stems in a scene and extract the fruit-bearing branch positions based on mask relationships. The location of the litchi cutting point was realized by introducing depth reference points and fruit stalk positioning lines. Lin et al. (2021) developed a new architecture for instance segmentation by using a tiny Mask R-CNN. It was trained with a small number of images and used to detect guava fruits and branches. The detected fruit and branch point clouds were fitted with spheres and cylinders. The proposed pipeline provided a robust method for branch detection and modeling under varying illumination environments. Tang et al. (2023) used an improved YOLOv4-tiny model to detect fruits, and the three-dimensional coordinates of fruit-picking points were located based on binocular stereo vision. These studies were applied to fruit picking. However, the segmentation method of fruit-bearing branches based on tree structure and the spatial location of picking points could provide theoretical support in identifying potential pruning points.

Díaz et al. (2018) used fast library for approximate nearest neighbor (FLANN), support vector machine (SVM), and density-based spatial clustering of applications with noise (DBSCAN) to locate grape buds in three-dimensional space, where FLANN was used to match key points, SVM was used to classify 3D points, and finally, DBSCAN was used to locate grape buds. Fernandes et al. (2021) used Mask R-CNN to identify potential pruning points of grape trees with a simple tree shape and finally determined the pruning locations of branches in combination with the graph theory algorithm. Adhikari et al. (2011) and Karkee et al. (2014) used the medial axis thinning algorithm to generate the three-dimensional skeleton of the apple tree point cloud and recognized pruning points under the guidance of the pruning rules. This method required a group of high-density point clouds; otherwise, it easily causes abnormal branches in the skeleton. Medeiros et al. (2017) proposed a robust method based on Yan et al. (2009) to avoid searching the tree skeleton directly in a point cloud. This method constructed local point cloud clusters through segmentation and clustering and then used the minimum spanning tree algorithm to connect the centroids of clusters to form a geometric skeleton. Finally, the locations of the pruning point were identified according to the trunk characteristics and the connection relationship between the primary branches and the trunk. However, when the branch structure was complex and multiple branches overlapped, the results of the minimum spanning tree algorithm were often unpredictable, such as the shape of disconnected branches or abnormally connected branches. Elfiky et al. (2015) realized 3D reconstruction based on the geometric features of a skeleton and used a new adaptive circle-based-layer-aware modeling scheme to locate pruning points, with a recognition accuracy of 96%. The above studies have explored the detection, segmentation, and identification of pruning points, but they mainly focused on the apple trees with trunk-shaped and grape trees with simple tree structures abroad, which are not suitable for the identification of pruning points of jujube trees.

In order to realize the automatic selection pruning of open-center jujube trees, we presented a method of extracting the jujube tree skeleton and identifying pruning points based on the space colonization algorithm. This paper studied acquiring multi-angle point cloud images of jujube trees by building a three-dimensional information acquisition platform and registering point clouds to achieve a three-dimensional reconstruction of jujube trees. Due to the large number of point clouds and their insignificant features, the space colonization algorithm iteratively generated tree skeletons through local point clouds to describe the three-dimensional shape of jujube trees. Based on the tree skeleton, a directed graph was constructed to form a geometric skeleton to represent the relationship between skeleton points. Pruning points were identified by the skeleton analysis algorithm and the pruning rule.

## 2 Data materials

The experimental site was the jujube garden (81°28′E, 40°34′N) of the 13th Regiment of Alar, Xinjiang. In the garden, perennial dwarf and densely planted open-center jujube trees were planted, and the tree structure included a trunk, a primary branch, and a lateral branch. The trunk was vertical and short in length. The number of primary branches was three to four. The tree height was 1.5–1.9 m, the plant spacing was 1.5 m, and the row spacing was 4.2 m.

**Figure 1:**
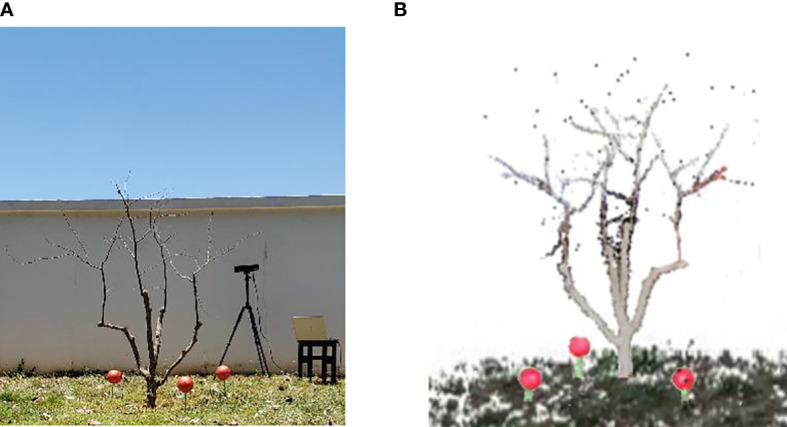
Data acquisition. **(A)** Experimental scene. **(B)** Original color point cloud image on one side.

The information collection platform built by the previous research was used to obtain leafless jujube tree information about 2 m from the tree trunk (**Figure 1A**). The information collection device was Kinect V2 (specifications are shown in **Table 1**), and its accuracy was 2 mm (Yang et al., 2015). Data were collected between 4 p.m. and 5 p.m. in the field. The sunlight was weak, and it was easy to obtain high-quality color images during the period. Kinect V2 was used to obtain the color images (1,920 × 1,080 pixels) and depth images (512 × 424 pixels) while registering the depth image to the color image to generate a 3D point cloud image (**Figure 1B**). The first visual view (0°) was named Tree_F, and the other view (counterclockwise rotation 180° of the system platform) was named Tree_B.

**Table 1:** General specifications of Kinect V2.

Feature	Specification	Value
Depth sensing	Basic principle Depth range Depth image resolution Field of view Frame rate	ToF 0.4–4.5 m 512 × 424 pixels 70° × 60° 30 Hz
Color camera	Color image resolution Frame rate	1,920 × 1,080 pixels 30 Hz (15 Hz in low light)
Active infrared	Infrared image resolution Frame rate Infrared light wavelength	512 × 424 pixels 30 Hz ∼827–850 nm
Data transmission	Interface standard	USB 3.0

## 3 Reconstruction of the dormant jujube tree

### 3.1 Point cloud preprocessing

**Figure 2:**
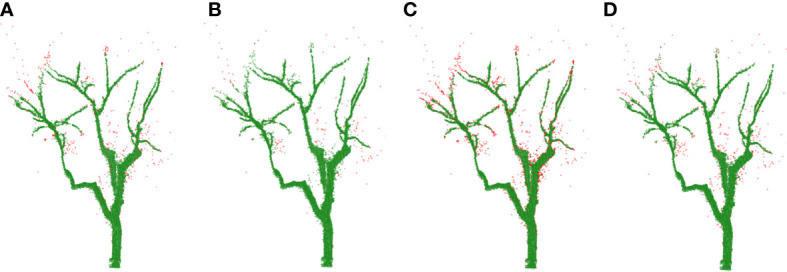
Comparisons of removing noise (noise is shown in red color). **(A)** *k* = 30, *λ* = 0.2. **(B)** *k* = 30, *λ* = 0.5. **(C)** *k* = 40, *λ* = 0.2. **(D)** *k* = 40, *λ* = 0.5.

Point cloud images obtained by the information acquisition system included ground point clouds, background noise, and outlier noise, so it was necessary to preprocess the original point cloud to obtain the point cloud information of a single jujube tree. According to the 3D point cloud coordinate information of jujube trees, by setting the distance threshold of the 3D point cloud of jujube trees, the point cloud less than the threshold was regarded as the inner point, and the point cloud of jujube trees outside the threshold was removed as the invalid point so as to remove the ground point cloud and background noise to obtain the point cloud of a single jujube tree. The outliers distributed around the branches of jujube trees were filtered by Statistical Outlier Removal (Runions et al., 2005). In the algorithm, parameter *λ* was the scaling factor, which was determined according to the point cloud density, and the empirical value was [0, 1]. The parameter *k* was the number of neighbors. In the process of parameter selection, excessive noise removal occurred in **Figures 2A, C**, and some branches were treated as noise removal. In **Figure 2B**, some noise was not removed. The best denoising effect is shown in **Figure 2D**.

### 3.2 Reconstruction pipeline

**Figure 3:**
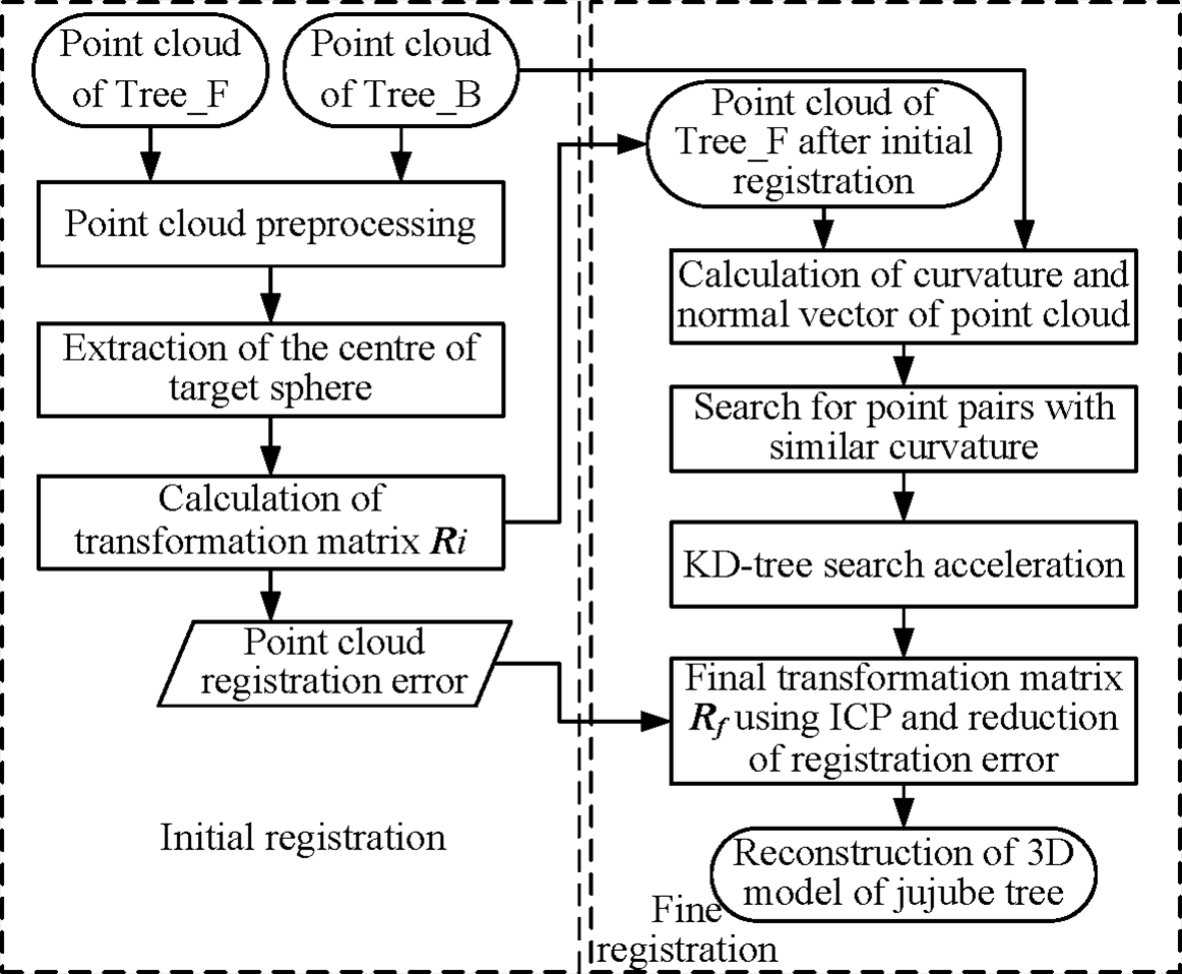
The overall reconstruction pipeline.

Point cloud data obtained under one-sided vision could not present the complete shape of jujube trees due to partial point cloud loss due to the occlusion between branches. The point clouds on both sides were registered through the information collection of two stations to make up for the lack of local point clouds under a single view (Geng et al., 2015). The Iterative Closest Point (ICP) algorithm (Besl and McKay, 1992) was widely used in the registration of images and point clouds. The registration process could be well completed when the spatial positions of the point clouds on both sides were close or the overlapping area was large, but the registration stability was poor when the point cloud positions were far away, and it was easy to fall into local optimization. The overall three-dimensional reconstruction process is described in **Figure 3**. First, the sphere centers of target balls in point clouds on both sides were extracted, and the transformation matrix ***R*** *_i_* was calculated based on the triangle similarity principle. The initial registration of point clouds on both sides was completed to make them close in space. The curvature of the point cloud was calculated based on the initial registration point cloud, and then the point pairs with similar curvature were searched. Finally, the ICP algorithm was used to obtain the transformation matrix ***R*** *_f_* to achieve fine registration. More details about the methods and algorithms used in the 3D reconstruction of dormant jujube trees were available in previous work (Fu et al., 2020).

## 4 Skeletonization based on space colonization algorithm

**Figure 4:**
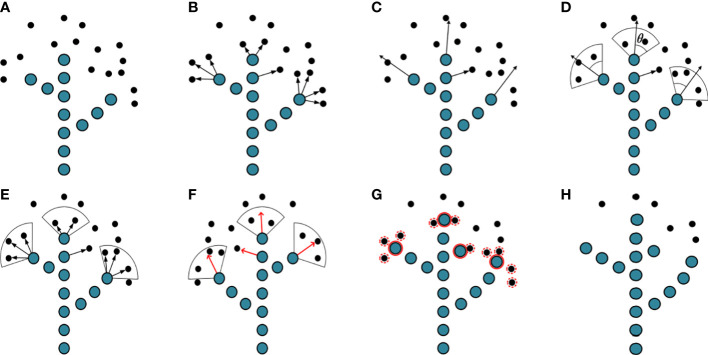
Illustration of space colonization algorithm. **(A)** Skeleton points and space points. **(B)** Connect the skeleton point with its space points. **(C)** Estimate neighborhood directions. **(D)** Calculate growth neighborhood. **(E)** Connect space points within growth neighborhoods. **(F)** Calculating growth vectors. **(G)** Generate new skeleton points and delete space points. **(H)** Complete an iteration.

The reconstructed point cloud model had a large amount of data and insignificant features, which is not conducive to tree structure analysis. It was necessary to transform the point cloud into a form that was easy to process so as to reduce information redundancy. The space colonization algorithm (Runions et al., 2005) was based on the principle of space competition for plant growth. The skeleton generated a tree-like structure by continuously competing for the growth space, which made the point cloud model into manageable skeleton data. In the process of skeleton generation, the neighboring space points of skeleton points influence the skeleton trend to be formed. The skeleton expanded continuously with the generation of skeleton points until the algorithm ended when no neighboring space points influenced the skeleton. **Figure 4** shows the skeleton generation process of the space colonization algorithm.

1) In **Figure 4A**, the current skeleton is composed of 13 blue skeleton points. There are 16 black space points *q* waiting to be searched globally to influence the skeleton trend. Initialize the tree skeleton array to store the tree skeleton coordinates. Assume that the search radius of the skeleton point *p* is *R*, and the space points *q* within the search radius are stored in set *S*(*p*). One space point *q* affects only the nearest skeleton point *p*, while the skeleton point *p* is affected by multiple space points within the search radius. The mathematical relationship between the skeleton point *p* and the space point in set *S*(*p*) is shown in Eq. (1).


(1) $$\left\{ \begin{array}{l} \left|q-p\right|<R\\ \left|p-q\right|=\min\left\{\left|p-q_{x}\right|,q_{x}\in S\left(p\right)\right\} \end{array}\right.$$

where *q* is the space point within the search radius *R* of the skeleton point *p*. *q_x_* is the serial number of space point *q* in *x* sets.

2) Estimate neighborhood directions. Connect the skeleton point with its space points in set *S*(*p*) as shown in **Figure 4B**. Calculate the direction vector, and use the sum of the direction vectors as the direction vector ***V_p_*** of the neighborhood, as shown by the black arrow in **Figure 4C**. ***V_p_*** is calculated by Eq. (2).


(2) $$V_{p}=\sum_{p\in S\left(p\right)}\frac{p-q}{\left\|p-q\right\|}$$

3) The neighborhood range is determined by the direction vector of the neighborhood. The growth neighborhood is a sector area with an angle of 2*θ* and a sector radius of *R*. The space points belonging to the neighborhood in set *S*(*p*) are calculated by Eq. (3). The space points belonging to the neighborhood are shown in **Figure 4D**.


(3) $$\arccos\left(V_{pq}\cdot V_{p}\right)\leq \theta$$

where ***V_pq_*** is the vector ***pq***.

4) Calculate the direction of child skeleton points generated by the current skeleton point (parent skeleton point). Connect the skeleton point to multiple space points in its growth neighborhood and calculate their respective vectors (vector direction from skeleton point to space point), as shown by the black arrow in **Figure 4E**. Take the direction of the vector sum as the growth direction of the skeleton and normalize its value, as shown by the red arrow in **Figure 4F**. The specific mathematical relationship is shown in Eq. (4).


(4) $$\{{\;}_{f=\frac{F}{\left\|F\right\|}}^{F=\sum_{p\in S\left(p\right)}\frac{p-q}{\left\|p-q\right\|}}$$

where ***F*** is the sum of vectors; ***f*** is the sum of normalized vectors.

5) The space distance between the skeleton point and the child skeleton point generated is *D_s_*. The position *P_i_* of the new skeleton point is calculated by Eq. (5).


(5) $$P_{i}=D_{s}\times f$$

6) The respective Euclidean distances between the child skeleton point and space points are calculated by Eq. (6). If the distance is less than the preset deletion threshold *R_d_*, the space point is deleted. The red solid circles are the child skeleton points participating in the calculation, and the red dashed circles are space points to be deleted, as shown in **Figure 4G**. In one iteration of the algorithm, two primary branches are expanded, the trunk grows upwards, and a new primary branch is formed, as shown in **Figure 4H**.


(6) $$\left|q-P_{i}\right|\leq R_{d}$$

**Figure 5:**
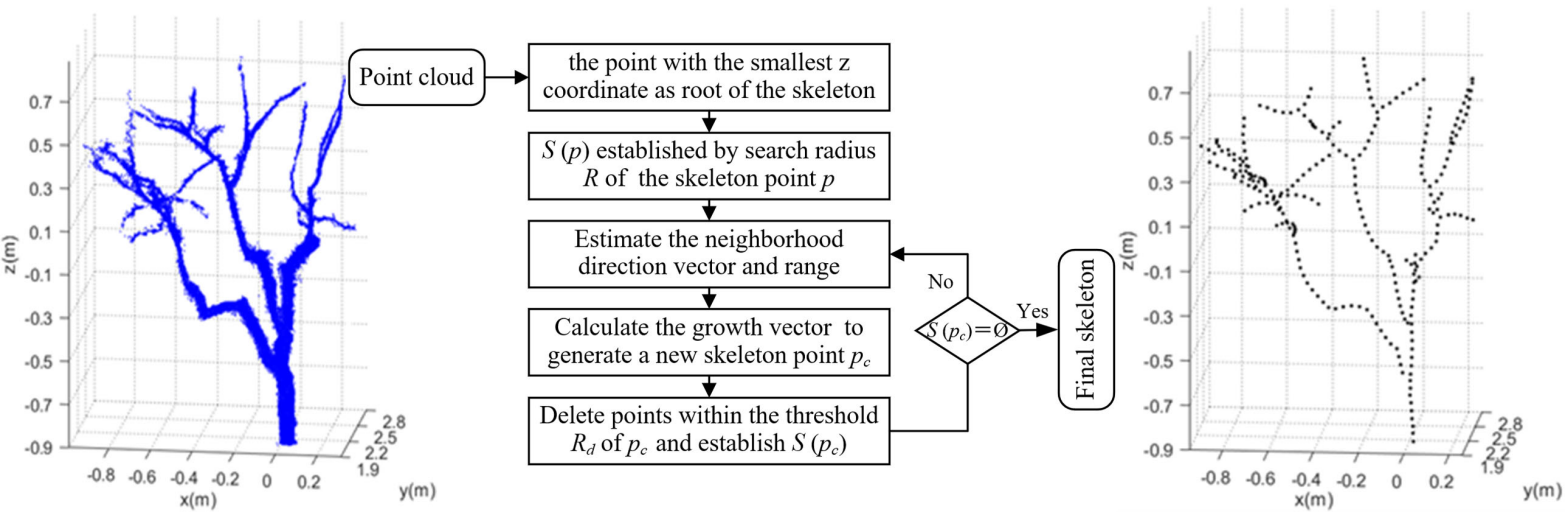
Flowchart of skeletonization using space colonization algorithm.

The space colonization algorithm took the reconstructed point cloud of a jujube tree as input and then iteratively generated the skeleton, as shown in **Figure 5**. The skeleton was a tree-like structure that reflected the topology of jujube trees. The skeleton point data were stored in the child linked list structure, and each parent skeleton point can query the generated child skeleton points.

## 5 Skeleton analysis and modeling

### 5.1 Trunk and primary branch identification

According to the generation relationship between skeleton points, the parent skeleton point connected its child skeleton points to form a directed graph *G*(*C*,*E*), and the direction was from the parent skeleton point to the child skeleton point, where $C={\left\{c_{i}\right\}}_{i=1}^{N_{c}}$ was the vertex set; that is, all skeleton points and *E* = {(*c_i_*, *c_k_*)} were the set of edges between connected skeleton points.

**Figure 6:**
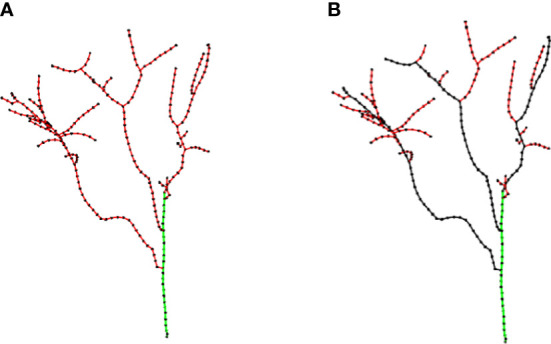
Results of skeleton analysis. **(A)** Trunk (green edges). **(B)** Primary branches (black edges).

After the directed graph *G* (*C*, *E*) was established, we needed to identify the subgraph *G_T_* (*C_T_*, *E_T_*) that corresponded to skeleton points that belong to the trunk. Since we knew that the trunk of the tree began close to the ground and followed an approximately vertical trajectory, we identified *G_T_* according to the following heuristics. Assume that initially *C_T_* = Ø and *E_T_* = Ø. The edge *e_i_* was the successor edge of *e_s_*. *e_i_* = {(*a*,*b*)|*a* = *c_i_*, and (*a*,*b*)∈*E*} was the set of edges in *E* that includes *c_i_* as one of its vertices. The beginning edge of the trunk was formed by the root skeleton point *c_i_* with the smallest *z* coordinate and its child skeleton points. Subsequently, the successor edges and the successor vertices were searched from the beginning edge. If *e_i_* was the only successor edge of *e_s_*, the edge *e_i_* = (*c_i_*, *c_k_*) was directly added to *E_T_*. When there were multiple successor edges, the change angle of *e_i_* relative to *e_s_* was calculated by Eqs (7) and (8) to determine the unique successor edge *e_i_*.


(7) $$\psi (\vec{e_{i}},\vec{e_{s}})=\cos^{-1}\left(\frac{\vec{e_{i}}\cdot \vec{e_{s}}}{\left\|\vec{e_{i}}\right\|\left\|\vec{e_{s}}\right\|}\right)$$

(8) $$e_{i}^{*}=\arg\min\left[\psi \left(\vec{e_{i}},\vec{e_{s}}\right)\right]$$

where $e_{i}^{*}$ corresponds to the successor edge that showed the smallest change angle relative to *e_s_* in *e_i_*. If *ψ* was within the threshold, $e_{i}^{*}$ = (*c_i_*, *c_k_*) was added to *E_T_*, and *c_i_* and *c_k_* were added to *C_T_*. Restart the procedure from *c_k_*, and terminate it when *ψ* exceeded the threshold. This approach guaranteed that the algorithm did not continue to search toward the primary branch at the top of the trunk. The green edges in **Figure 6A** show the result of the trunk detection algorithm.

Primary branches *G_B_* (*C_B_*, *E_B_*) were identified on the basis of trunk *G_T_* (*C_T_*, *E_T_*). The beginning point of the primary branch was the point belonging to the trunk, which was the vertex of the trunk with multiple edges or at the top of the trunk. For the convenience of description, the beginning points of the two types were collectively called *PB_start_*. After the trunk was identified, a depth-first search was used to find primary branches, as follows.

(1) Mark a beginning point *PB_start_* as the current point, and search the successor vertices of the current point as candidate points.
(2) Find an adjacent point not marked as a trunk among candidate points and mark the new point as the current point.
(3) The depth-first search is performed on the subgraph with the current point as the root node in the directed graph, and the vertices without successor points in the access process are marked as the endpoints.
(4) Trace back from the endpoint to the starting point *PB_start_* and calculate the sum of Euclidean distances *L_i_* between all the points in each backtracking path.
(5) Mark the path with the highest cumulative Euclidean distances as the primary branch beginning from *PB_start_*. Repeat steps 1 through step 5 until all primary branches are identified.

**Figure 6B** shows the final result of primary branch identification. The black edges were primary branches identified, and the remaining red edges were lateral branches.

### 5.2 Pruning point identification

**Figure 7:**
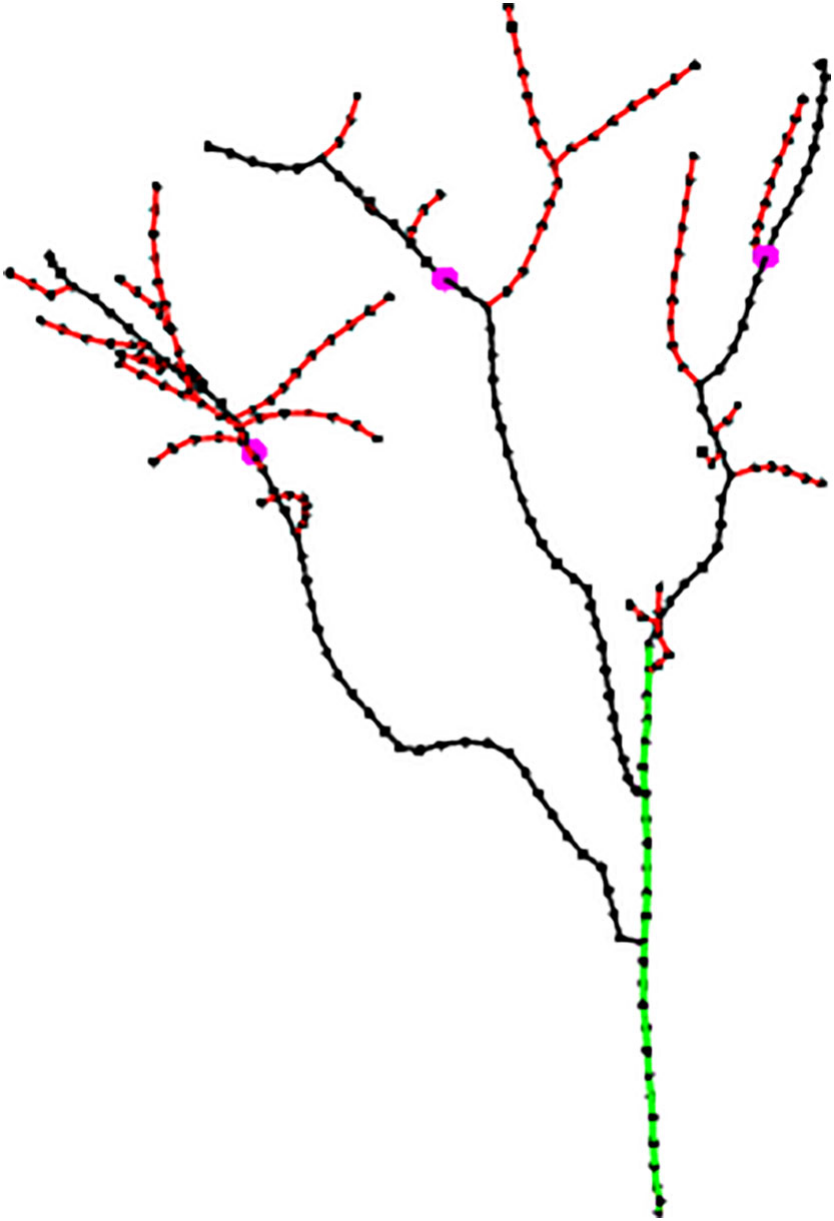
Identification results of pruning points (pink dots) on primary branches.

During the dormancy period, the managers cut off the end of the primary branch, which accounted for about 1/3 of the length of the primary branch. Pruning was helpful to reduce nutrient consumption in the dormancy period and make nutrient supply concentrated in the middle of main branches to cultivate fruiting branch groups so as to fully improve the production potential of jujube trees. Pruning rules, while simple, could greatly increase efficiency in seasonal pruning. Therefore, the length of the primary branch became the key parameter in the pruning point identification algorithm. In this study, the pruning length of the primary branch was not set to a fixed value but to an adaptive length, which located the pruning point at 2/3*L_i_* from the beginning point of the primary branch. The pink dots are the identified pruning points, as shown in **Figure 7**.

### 5.3 Modeling

**Figure 8:**
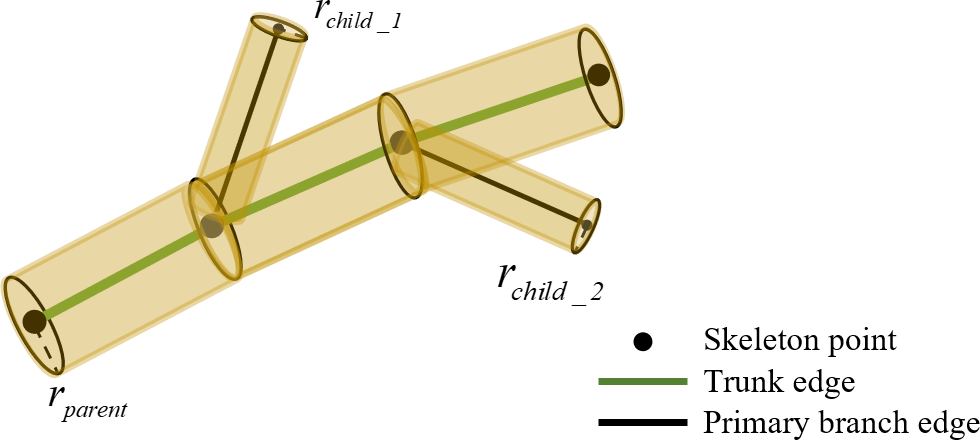
Schematic diagram of branch modeling.


To visualize the pruned jujube trees, the model was based on the pruned skeleton. The trunk of jujube trees was Grade 1 branch, the primary branches were Grade 2 branches, and the remaining branches were Grade 3 branches. The branch thickness gradually decreased from Grade 1 to Grade 3. The change of branch thickness followed the rule of gradually thinning from the beginning to the end. After the determination of the pruned skeleton information, the geometric model of the trunk and primary branches was represented by a multi-segment cylindrical connection. The edges in the directed graph were set as the center axis of the cylinder, as shown in **Figure 8**. However, the two base radii of the cylinder, that is, the thickness of the branch, have not been determined. The pipeline model theory was widely used in estimating branch thickness (Shi et al., 2019). The thickness of the parent branch and the generated child branches was calculated by Eq. (9).

(9) $$r_{parent}^{n}=\sum_{i=1}^{m}r_{child\_i}^{n}$$

where *r_parent_* is the beginning radius of the parent branch, *r_child_i_* is the beginning radius of the child branch, *m* is the number of child branches, and *n* is generally taken as 2. The root of the jujube tree was measured, and the beginning radius of all branches was calculated from the root. According to the pruned skeleton and radius, the cylinder was continuously generated along the skeleton trend to build a geometric model.

## 6 Results and analysis

### 6.1 Comparative analysis of point cloud registration errors

**Figure 9:**
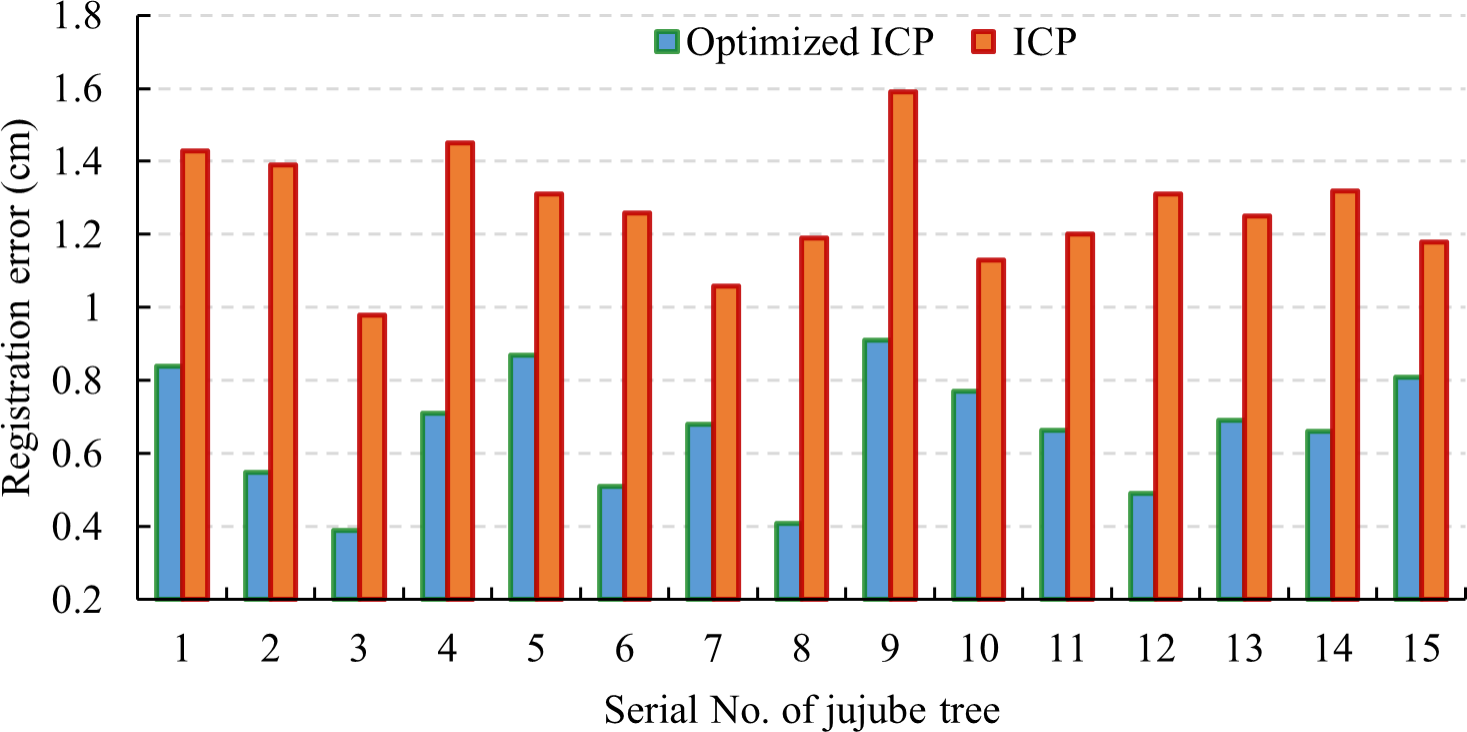
Comparison of point cloud registration errors.

To verify the stability of the individual reconstruction algorithm proposed in this study, 15 jujube trees were registered, and the results are shown in **Figure 9**. It could be seen that the registration errors were less than 0.91 cm, and the average registration error was 0.66 cm. The optimized ICP algorithm greatly improved the accuracy of point cloud registration. The comparison results show that the algorithm proposed in this study has high stability and reliability and meets the application requirements of a reconstructed jujube point cloud.

### 6.2 Skeleton extraction and pruning point identification

**Figure 10:**
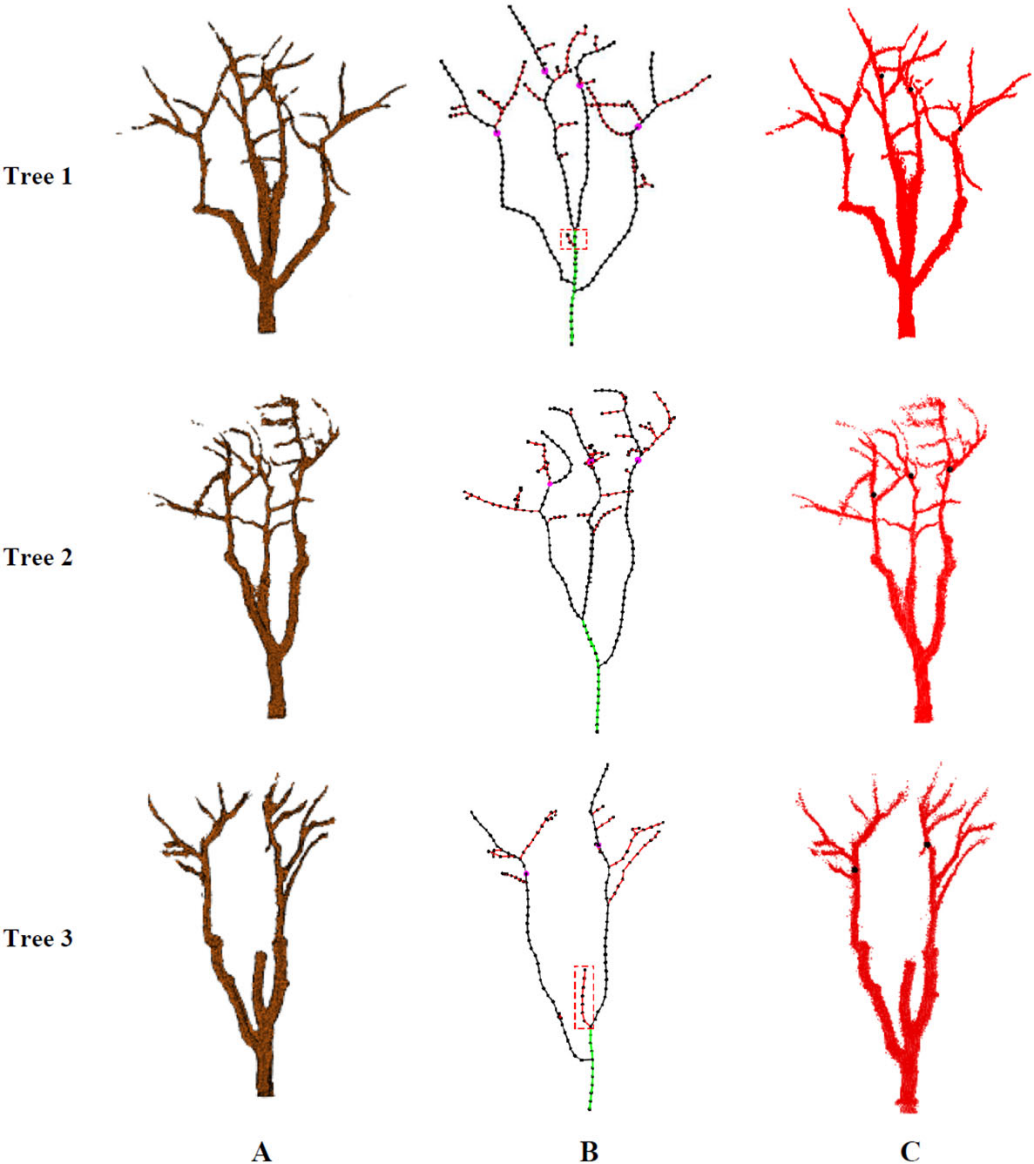
Results of skeleton analysis and pruning points identification. **(A)** Model of jujube tree. **(B)** Identification of pruning points based on skeleton analysis. **(C)** Mapping of pruning point positions in reconstructed point cloud.

The point cloud of jujube trees collected in the field was reconstructed to obtain complete point cloud information on jujube trees, and the Alpha-shape algorithm was used to triangulate the point cloud to improve the visual effect of the jujube tree model. The jujube tree individual model is shown in **Figure 10A**. A space colonization algorithm was used to extract the jujube tree skeleton with a search radius *R* of 8.2 cm, a growth neighborhood *θ* of 22.5°, a skeleton point spacing *D_s_* of 4.0 cm, and a deletion threshold *R_d_* of 6.0 cm. **Figure 10B** shows the skeleton generated by the space colonization algorithm, trunk and primary branches identified by the skeleton analysis algorithm, and pruning points located by the pruning rule. The number of beginning points *PB_start_* determined by the skeleton analysis algorithm in Tree 1 was four, of which three were the inner points of the trunk and one was the top endpoint of the trunk. Each inner point generated one primary branch, and the top point generated two primary branches. The red edge marked was the false primary branch because this beginning point was close to the bifurcation of two primary branches, and there was local noise at the same time, which resulted in unreasonable branches when the space colonization algorithm generated the skeleton. The red edge marked in Tree 3 has stopped growing due to necrosis, and its length was short, which did not meet the requirements of pruning. It was also one of the false primary branches. In order to improve the robustness of the algorithm to the identification of primary branches, the minimum length of primary branches was set to 50 cm so as to avoid adding false branches such as unreasonable branches or short branches to the primary branches *G_B_* (*C_B_*, *E_B_*). After the length threshold setting, the global skeleton was marked as the trunk (green edges), primary branches (black edges), and lateral branches (red edges) by the algorithm. Finally, pruning points (pink dots) of primary branches were identified under the guidance of the pruning rule.

In order to evaluate the reliability of the pruning point information and ensure that the robot could achieve the pruning operation according to the pruning points, the three-dimensional coordinate information of the pruning points was mapped in the reconstructed point cloud. Pruning point information in **Figure 10C** was located on the primary branches, which showed that the identified pruning point information was effective, and also verified that the skeleton extracted by the space colonization algorithm had a high degree of coincidence with the three-dimensional structure of jujube trees.

### 6.3 Modeling

**Figure 11:**
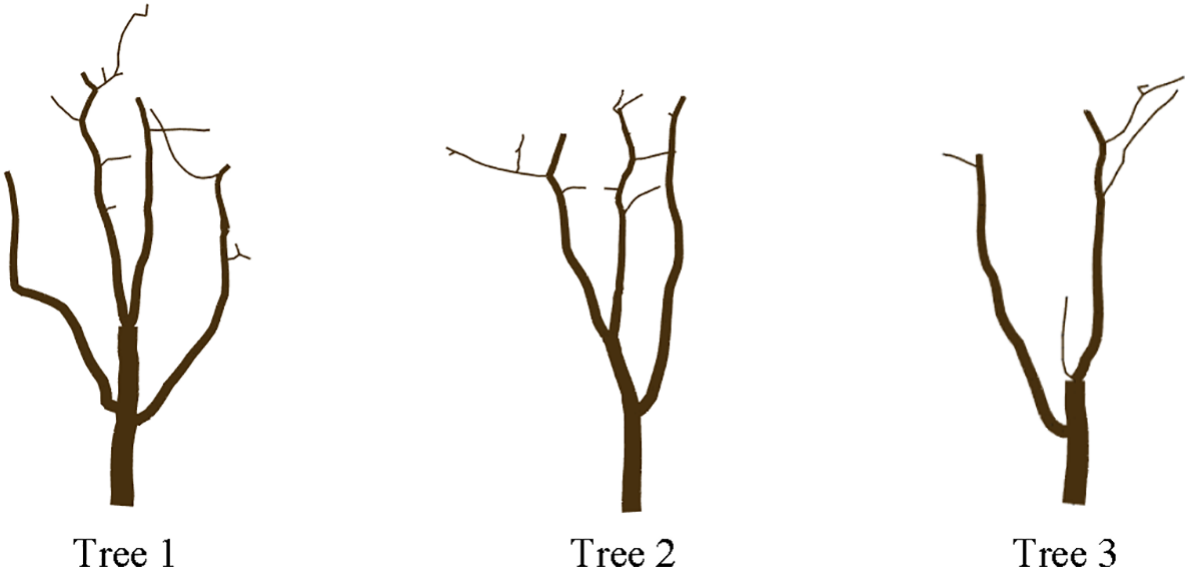
Model of jujube tree after pruning.

Branch modeling could predict the shape of jujube trees after pruning in advance so as to avoid irreversible operations such as incorrect pruning. According to the actual pruning operation, the pruned branch was partially removed from the tree, that is, all skeleton points in the skeleton generated by the pruning point were deleted. The root radii of Tree 1, Tree 2, and Tree 3 were measured at 4.1, 3.4, and 4.0 cm, respectively. The beginning radius of all branches was calculated from the root. The roughness of the beginning of all branches was calculated from the bottom to the top of the root, and the visualization of the pruned jujube tree was achieved by combining the central axis information of the pruned skeleton (**Figure 11**).

## 7 Discussion

**Figure 12:**
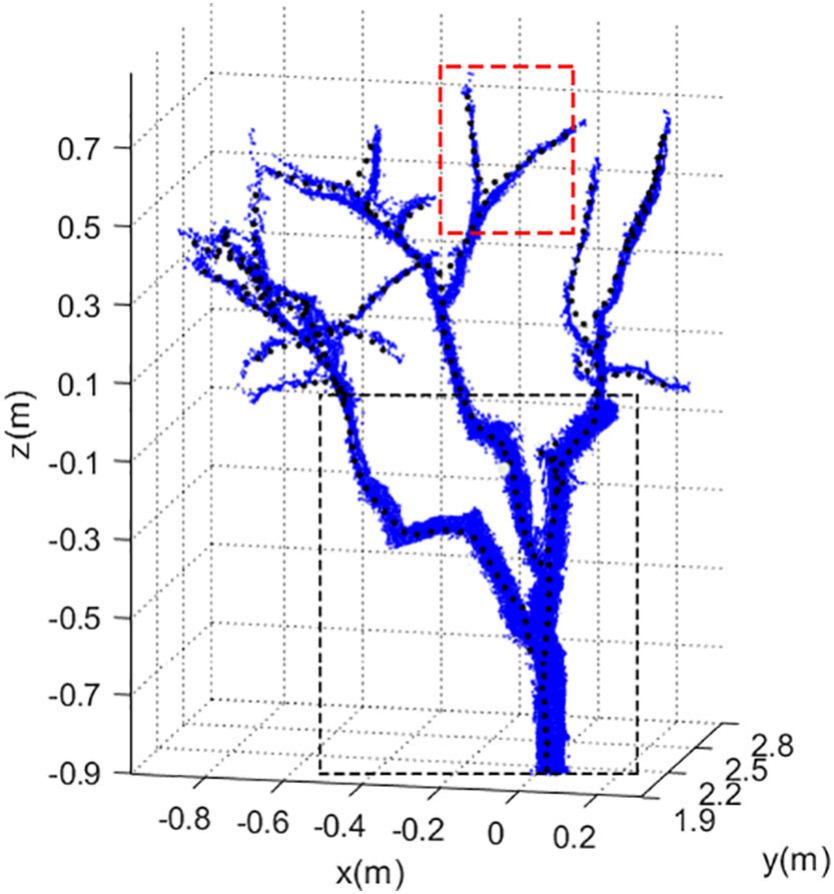
Point cloud of jujube tree (blue) and its skeleton (black).

The final shape of the tree skeleton depended on four parameters for search radius, growth neighborhood, skeleton point spacing, and deletion threshold of the space colonization algorithm. In the process of skeleton generation by the algorithm, the skeleton of the trunk and primary branches best matched the tree shape as shown in the black box (**Figure 12**). The skeleton at the bifurcation of the primary branch and the lateral branch in the red box deviated from the point cloud but returned to normal quickly. This was because when the space colonization algorithm iteratively generated the lateral branch, the search radius contained more point clouds of the primary branch, which interfered with the skeleton generation of the lateral branch. However, as the distance between the lateral branch and the primary branch increased, the number of point clouds belonging to the primary branch decreased. Subsequently, the influence of the primary branch was weakened, and finally, the lateral branch skeleton returned to normal and coincides with the tree shape.

The search radius of the algorithm was robust when generating trunk and primary branches. However, for the lateral branches, if the search radius was too large, it would be easily affected by primary branches. Therefore, when formulating the pruning rule, pruning points were not determined by the fixed pruning length but selected 1/3 of the distance from the end of the primary branch to avoid the place where the side branches were dense as far as possible and located pruning points in the robust area of the skeleton. In the next work, the adaptability of parameters should be improved. With the change in branch density, the search radius and deletion threshold should be appropriately increased or reduced to further improve the similarity between skeleton and tree shape and ensure the effectiveness of identifying pruning points.

## 8 Conclusions

In this study, a low-cost depth camera was used to collect point cloud data on both sides of jujube trees. The point cloud data were preprocessed to remove noise. Point cloud registration was conducted by combining manual marking with an optimized ICP algorithm to achieve the reconstruction of jujube individual information. The registration errors were less than 0.91 cm, and the average registration error was 0.66 cm, which provided good individual data for the skeleton generation of the space colonization algorithm. Triangulate the reconstructed point cloud with the Alpha-shape algorithm, which could make the model more similar to the real tree in appearance, and was the visual basis for the automatic pruning of jujube trees.

The space colonization algorithm generated the skeleton reflecting the topological structure of the jujube tree by iteration. A directed graph was constructed using skeleton points to represent the tree structure and the parent–child relationship between skeleton points. According to the tree structure and the characteristics of the directed graph of jujube trees, the skeleton was analyzed to identify the trunk and primary branches. The branch length threshold solved the problem of false primary branches in the skeleton. Subsequently, under the guidance of the pruning rule, the pruning points were identified, and the validity of the pruning points was verified by using the information mapping method. Finally, the visual model was constructed based on the remaining skeleton information to realize the appearance of the pruned tree.

Primary branches of jujube trees identified by the algorithm might deviate from the result of our actual judgment because the branch length obtained by the depth-first search was the only factor for primary branch identification. In the next step, we will take into account both the length of the main branch and the angle change of the main branch at the branch bifurcation and add appropriate weights to these two factors. We will sort the possible primary branches originating at the same point through the scoring principle so that the results of the algorithm are more consistent with human judgment.

## Data availability statement

The original contributions presented in the study are included in the article/supplementary material. Further inquiries can be directed to the corresponding authors.

## Author contributions

YF designed and performed the experiment, selected the algorithms, analyzed the data, debugged the algorithms, and wrote the manuscript. YF, YX, MF, YW, and CS collected the data. HZ monitored the data analysis. WF and HZ conceived the study and participated in its design. All authors contributed to this article and approved the submitted version.
